# Adding a single low-dose of primaquine (0.25 mg/kg) to artemether-lumefantrine did not compromise treatment outcome of uncomplicated *Plasmodium falciparum* malaria in Tanzania: a randomized, single-blinded clinical trial

**DOI:** 10.1186/s12936-016-1430-3

**Published:** 2016-08-26

**Authors:** Richard Mwaiswelo, Billy Ngasala, Irina Jovel, Berit Aydin-Schmidt, Roland Gosling, Zul Premji, Bruno Mmbando, Anders Björkman, Andreas Mårtensson

**Affiliations:** 1 Department of Parasitology and Medical Entomology, Muhimbili University of Health and Allied Sciences, Dar Es Salaam, Tanzania; 2 Department of Microbiology, Tumor and Cell Biology, Karolinska Institutet, Stockholm, Sweden; 3 Department of Epidemiology and Biostatistics, University of California San Francisco, San Francisco, CA USA; 4 Global Health Group, University of California San Francisco, San Francisco, CA USA; 5 Aga Khan University Hospital, Nairobi, Kenya; 6 Tanga Centre, National Institute for Medical Research, Tanga, Tanzania; 7 Department of Women’s and Children’s Health, International Maternal and Child Health (IMCH), Uppsala University, Uppsala, Sweden

**Keywords:** *Plasmodium falciparum* malaria, Artemether-lumefantrine, Primaquine, Cure rate

## Abstract

**Background:**

The World Health Organization (WHO) recently recommended the addition of a single low-dose of the gametocytocidal drug primaquine (PQ) to artemisinin-based combination therapy (ACT) in low transmission settings as a component of pre-elimination or elimination programmes. However, it is unclear whether that influences the ACT cure rate. The study assessed treatment outcome of artemether-lumefantrine (AL) plus a single PQ dose (0.25 mg/kg) versus standard AL regimen for treatment of acute uncomplicated *Plasmodium falciparum* malaria in Tanzania.

**Methods:**

A randomized, single-blinded, clinical trial was conducted in Yombo, Bagamoyo district, Tanzania. Acute uncomplicated *P. falciparum* malaria patients aged ≥1 year, with the exception of pregnant and lactating women, were enrolled and treated with AL plus a single PQ dose (0.25 mg/kg) or AL alone under supervision. PQ was administered together with the first AL dose. Clinical and laboratory assessments were performed at 0, 8, 24, 36, 48, 60, and 72 h and on days 7, 14, 21, and 28. The primary end-point was a polymerase chain reaction (PCR)-adjusted adequate clinical and parasitological response (ACPR) on day 28. Secondary outcomes included: fever and asexual parasitaemia clearance, proportion of patients with PCR-determined parasitaemia on day 3, and proportion of patients with *Pf*mdr1 N86Y and *Pf*crt K76T on days 0, 3 and day of recurrent infection.

**Results:**

Overall 220 patients were enrolled, 110 were allocated AL + PQ and AL, respectively. Parasite clearance by microscopy was fast, but PCR detectable parasitaemia on day 3 was 31/109 (28.4 %) and 29/108 (26.9 %) in patients treated with AL + PQ and AL, respectively (*p* = 0.79). Day 28 PCR-adjusted ACPR and re-infection rate was 105/105 (100 %) and 101/102 (99 %) (*p* = 0.31), and 5/107 (4.7 %) and 5/8 (4.8 %) (*p* = 0.95), in AL + PQ and AL arm, respectively. There was neither any statistically significant difference in the proportion of *Pf*mdr1 N86Y or *Pf*crt K76T between treatment arms on days 0, 3 and day of recurrent infection, nor within treatment arms between days 0 and 3 or day 0 and day of recurrent infection.

**Conclusion:**

The new WHO recommendation of adding a single low-dose of PQ to AL did not compromise treatment outcome of uncomplicated *P. falciparum* malaria in Tanzania.

*Trial registration number* NCT02090036

**Electronic supplementary material:**

The online version of this article (doi:10.1186/s12936-016-1430-3) contains supplementary material, which is available to authorized users.

## Background

Artemisinin-based combination therapy (ACT) is generally recommended as first-line treatment for uncomplicated *Plasmodium falciparum* malaria globally [1]. Recently, the World Health Organization (WHO) has recommended the addition of a 0.25 mg/kg single-dose of the gametocytocidal drug primaquine (PQ) to standard ACT regimen as a component of pre-elimination or elimination of malaria in low-intensity transmission settings and for containment in areas threatened by artemisinin resistance [2, 3]. Most concerns with this new recommendation have been on safety, due to the potential risk of PQ-induced haemolysis in glucose-6-phosphate dehydrogenase (G6PD)-deficient patients. Equally important is to ensure that the treatment outcome of ACT is not compromised by the addition of a single-low dose of PQ. This is of particular concern since the individual patient with uncomplicated malaria does not personally benefit from PQ intake, the potential benefits, i.e., of reduced transmission, are rather on community level. However, no study has reported on the cure rate of ACT in addition to 0.25 mg/kg single-dose PQ.

Anti-malarial drug efficacy depends upon appropriate drug levels being reached and maintained for a long enough time for the drug to act [4]. Insufficient exposure is associated with increased risk of treatment failure. Inhibition of drug metabolism through drug–drug interaction may lead to insufficient exposure and consequently reduced efficacy [4, 5]. However, comprehensive data on potential interactions between artemether-lumefantrine (AL) and PQ are currently lacking, and therefore it remains unclear whether the addition of this single low-dose (0.25 mg/kg) PQ may compromise the efficacy of AL [6].

The aim of this study was to assess treatment outcome of the recent WHO recommendation of adding a single PQ dose (0.25 mg/kg) to AL versus standard AL regimen for treatment of acute uncomplicated *P. falciparum* malaria in Tanzania.

## Methods

### Study area

This trial was conducted at Yombo primary health facility, Bagamoyo district, Tanzania, between July and November, 2014. The health facility is located southwest, about 20 km, from Bagamoyo town. It serves approximately 7000 people and has capability to carry out routine malaria microscopy and rapid diagnostic test.

Malaria transmission is high and occurs throughout the year with peaks related to the long rain season from May to July and short rain season from November to December. In the study area, *P. falciparum* is the major malaria species and *Anopheles gambiae* sensu stricto the main vector [7–9]. AL has been used as the first-line treatment for uncomplicated malaria since 2006. Sulfadoxine-pyrimethamine is used for intermittent preventive treatment in pregnant women. Long-lasting, insecticide-treated mosquito nets is the major vector control method [10]. G6PD deficiency prevalence in the study area has previously been estimated to 13.6 % in hemizygous males and 4.5 % in homozygous females [11].

### Study design

This was a randomized, single-blinded, clinical trial comparing treatment outcome and safety of AL plus a single low-dose PQ (AL + PQ) versus standard AL regimen. Safety outcomes have been presented in a separate publication [11]. Patients with uncomplicated microscopically confirmed *P. falciparum* mono-infection were enrolled, randomly assigned to either AL + PQ or AL treatment, admitted during the first 3 days of treatment and thereafter followed up until day 28 after treatment initiation.

Treatment outcome was based on polymerase chain reaction (PCR)-adjusted parasitological cure. Therapeutic failures were classified as; early treatment failure (ETF), late clinical failure (LCF), or late parasitological failure (LPF) [12].

### Study population

Patients presenting at the study site with suspected acute uncomplicated malaria were screened for eligibility. Inclusion criteria were age ≥1 year, weight ≥10 kg, body temperature ≥37.5 °C or history of fever in the last 24 h, microscopy confirmed *P.falciparum* mono-infection, any parasitaemia level, ability to swallow oral medication, ability and willingness to abide by the study protocol and the stipulated follow-up visits, and a written informed consent (in case of children a proxy consent from a parent/guardian). Exclusion criteria were evidence of severe malaria or danger signs, known allergy to trial medicines, reported anti-malarial intake ≤2 weeks, haemoglobin (Hb) <8 g/dL, blood transfusion within last 90 days, febrile condition other than malaria, known underlying chronic or severe disease (including severe malnutrition), pregnancy and breastfeeding.

### Randomization and blinding

Treatment allocation was done using sex-stratified, block randomization with four blocks, two per treatment arm using RESEARCH RANDOMIZER (version 4) (computer software) (Wesleyan University, Connecticut, USA) [13].

Opaque envelopes containing the pre-determined treatment codes were kept serially in a male and female strata. The envelopes were opened by a study nurse just before the first treatment dose was administered. Patients were blinded to the assigned treatment.

### Treatment

A standard, weight-based, three-day course of AL (Coartem^®^, Novartis) was administered to all patients according to Tanzania national treatment guideline for uncomplicated *P. falciparum* malaria [14]. AL dispersible tablets suspended in water were administered to children who were not able to swallow tablets. A single 0.25 mg/kg PQ dose (Primaquine phosphate, Sanofi) was administered together with AL first dose to patients assigned AL + PQ treatment. The accuracy of PQ dose among patients weighing less than 60 kg body weight was ensured by administering the drug in aqueous solution, whereas for adults weighing 60 kg and above the drug was administered as tablet. To make aqueous solution, a 15 mg PQ tablet was suspended in 15 mL of water, and the dose was measured using a sterile syringe based on body weight. To achieve the single blinding, the PQ dose was prepared in the absence of patients, and a glucose-based syrup was added to mask the PQ taste, whereas for patients allocated AL alone, the same glucose-based syrup was administered with AL first dose. Adult patients allocated to the AL + PQ arm, during consenting, never knew in which treatment arm they were allocated, and during drug administration, PQ tablet was mixed with AL tablets, however, in case patients asked about the additional tablet, they were told that the tablet was given to prevent treatment side effects. In order to optimize AL absorption and minimize PQ gastro-intestinal side effects, biscuits were administered prior to all drug doses [15]. A study nurse administered/supervised intake of all drug doses. Patients were monitored for 30 min after each drug dose. Treatment was re-administered in case of vomiting within this period.

### Lost follow-up and patient withdrawal

Study withdrawal criteria were: vomiting the trial medicine >three times, withdrawal of consent, intake of any medicine with anti-malarial properties outside the study protocol, or any protocol violation. Subjects were considered lost to follow-up and consequently withdrawn if they missed a scheduled follow-up visit and did not attend on the successive days despite efforts to trace them at their homes [12]. A subject who returned before the last day of follow-up was not considered a lost follow-up. Subjects with symptoms/signs of severe disease (including repetitive vomiting of trial medicine) were treated according to the national guideline and were then followed up until recovery.

### Procedures

Clinical assessment was performed at 0, 8, 24, 36, 48, 60, and 72 h and on days 7, 10, 14, 21, 28 or on any day of recurrent illness. The assessment included history of clinical symptoms, possible adverse events, concomitant drug consumption and clinical examination including measurement of axillary temperature. Fever was defined as body temperature ≥37.5 °C. A case record form was used to record all clinical and laboratory data. Laboratory assessment involved collection of finger-prick blood samples for Hb concentration, thick smears for microscopy-determined asexual and sexual parasitaemia, thin smears for species determination (at enrolment) and filter-paper blood samples for parasite detection and genotyping by PCR. Hb assessment was done on days 0, 1, 2, 3, 7, 10, 14, 21, 28 or on any day of recurrent illness, whereas, filter paper and blood slide samples were collected at 0, 8, 24, 36, 48, 60, and 72 h and on days 7, 10, 14, 21, 28 or on any day of recurrent illness. Hb concentration was measured using a portable spectrophotometer, HemoCue Hb 201+ (HemoCue AB, Ängelholm Sweden), with a precision of ±0.3 g/dL. A control cuvette at 16.0 ± 0.3 g/dL was used for daily calibration according to manufacturer’s instruction.

A 10 % Giemsa solution was used to stain thick and thin blood smears. Thin smears were prepared once, i.e., at enrolment, whereas thick smears were prepared at all sampling time points. Asexual parasites were counted against 200 white blood cells (WBC). The obtained number was multiplied by 40, assuming 8000 leukocytes per μL of blood, to gain an approximate parasite count. In case no parasites were observed after examining 100 fields, the blood slide was considered negative. Each slide was read by two independent microscopists. In case they disagreed on presence of parasitaemia or if density differed by more than 25 %, a third independent reading was performed. In case of positive versus negative results, a third independent reading was used to confirm the reading of the first two readers. The filter paper (3MM Whatman) blood samples were labelled, air-dried at room temperature for 3–4 h and then packed in individual plastic bags and stored. After study completion, all filter papers were transported to Karolinska Institutet, Sweden, for molecular analysis.

### Molecular analysis

A 10 % Chelex-100^®^ method was used to extract genomic DNA from dried blood spots [16]. Paired blood samples (pre-treatment and day of recurrent parasitaemia) from patients classified as LCF or LPF were genotyped to differentiate recrudescence from re-infection by stepwise genotyping of *P. falciparum* block 3 of merozoite surface protein (msp) 2, block 2 of msp 1 and region II (RII) of glutamate-rich protein (glurp) [17]. The respective initial amplifications were followed by individual nested PCR reactions using family specific primers for msp1 (K1, MAD20 and RO33) and msp2 (FC27 and IC) and semi-nested for RII of glurp [17]. The amplicons were loaded on a GelRed™ (Biotium, Hayward, CA, USA) stained agarose gel, separated by electrophoresis and then visualized under ultraviolet transillumination (Gel Doc™, Bio-Rad, Hercules, CA, USA), and sized by Image Lab™ software (Bio-Rad, Hercules, CA, USA). Alleles in each family were considered the same if fragments size were within 20 base pair interval. Subjects with recurrent parasitaemia by microscopy, which could not be confirmed by PCR were considered to have uncertain-PCR-adjusted outcome.

Recrudescence was defined as presence of at least one matching allelic band, and re-infection was defined as absence of any matching allelic band at baseline and on the day of parasite recurrence [17]. In addition*, P. falciparum* multi-drug resistant gene 1 (*Pf*mdr1) asparagine (N)-86-tyrosine (Y) and chloroquine resistance transporter gene (*Pf*crt) lysine (K)-76-threonine (T) were genotyped using nested PCR followed by restriction fragment length polymorphism using *Apo*I restriction enzyme as previously described [18].

### Study end-points

The primary outcome was the proportion of patients with PCR-adjusted adequate clinical and parasitological response (ACPR) by day 28. Secondary outcomes included: fever and asexual parasite clearance, proportion of patients with PCR-determined parasitaemia on day 3, PCR- determined re-infection rate, and proportion of patients with *Pf*mdr1 N86Y and *Pf*crt K76T on days 0, 3 and day of recurrent infection.

### Statistical analysis

Sample size calculation was based on equivalence, defined as margin of 10 % PCR-adjusted ACPR, between the treatments. Allowing for 10 % attrition, 80 % power at 0.05 significance, a sample size of 110 per treatment arm was required. Data were double-entered in an electronic database and analysed using SPSS software version 16 (SPSS Inc, Chicago, USA) as per protocol. Independent sample *t* test was used to compare means. Non-parametric data were compared using Chi square, Fisher’s or McNemar tests as appropriate. Cure rate end points were analysed by survival analysis, and the survival curves of the two treatment arms were plotted and compared using log-rank test. Data were censored at the time of withdrawal for patients lost to follow up, withdrew consent and PCR determined re-infection or uncertain PCR outcome. A *p* ≤ 0.05 was considered significant.

## Results

### Patients’ characteristics and trial profile

**Fig. 1:**
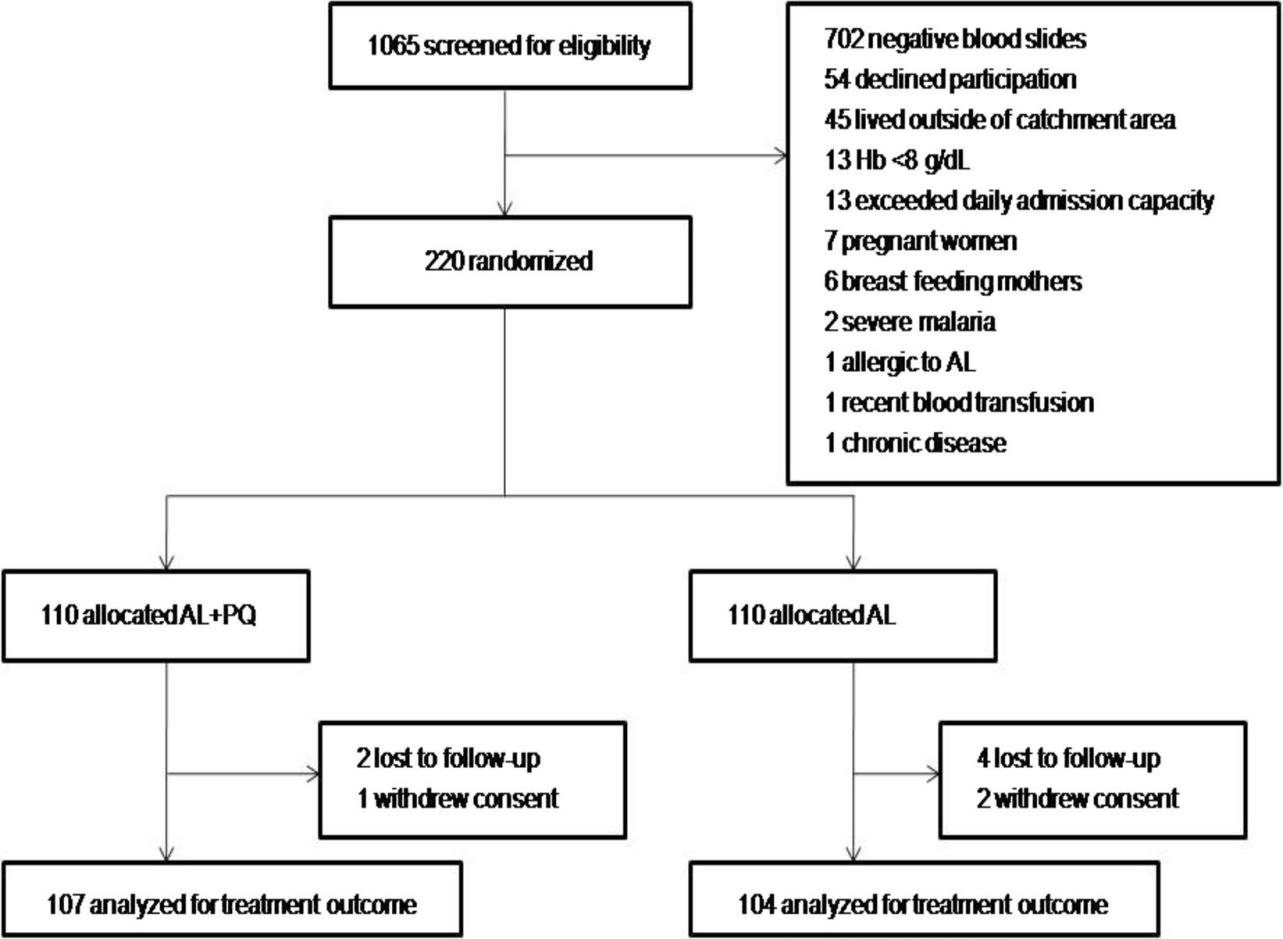
Flow of patients through the trial

The flow of patients through the trial is presented in Fig. 1. In summary, a total of 1065 subjects were screened for eligibility, of whom 845 were not included. The remaining 220 were enrolled, 110 per treatment arm. Pre-treatment characteristics of the study participants are presented in Table 1.

**Table 1:** Pre-treatment characteristics of the study subjects

Characteristic	Treatment arm		*P*-value
	AL + PQ	AL	
	n = 110	n = 110	
Female, n (%)	55 (50)	55 (50)	1.0
Age (years), median (range)	9 (5.6–15)	10 (5–23)	0.392^‡^
Body weight (kg), mean (SD)	30.7 (17.1)	34.3 (19.6)	0.145^†^
Body temperature (°C), mean (SD)	38.3 (1.1)	38.3 (1.3)	0.977^†^
Fever (≥37.5 °C), n (%)	88 (80)	80 (73)	0.204^¥^
Geometric mean asexual parasite density/µl (95 % CI)	8327 (5451–12,717)	8384 (5437–12,927)	0.982^†^

^‡^Mann–Whitney test, ^†^ Student t-test, ^¥^ Chi square test

### Treatment outcomes

**Fig. 2:**
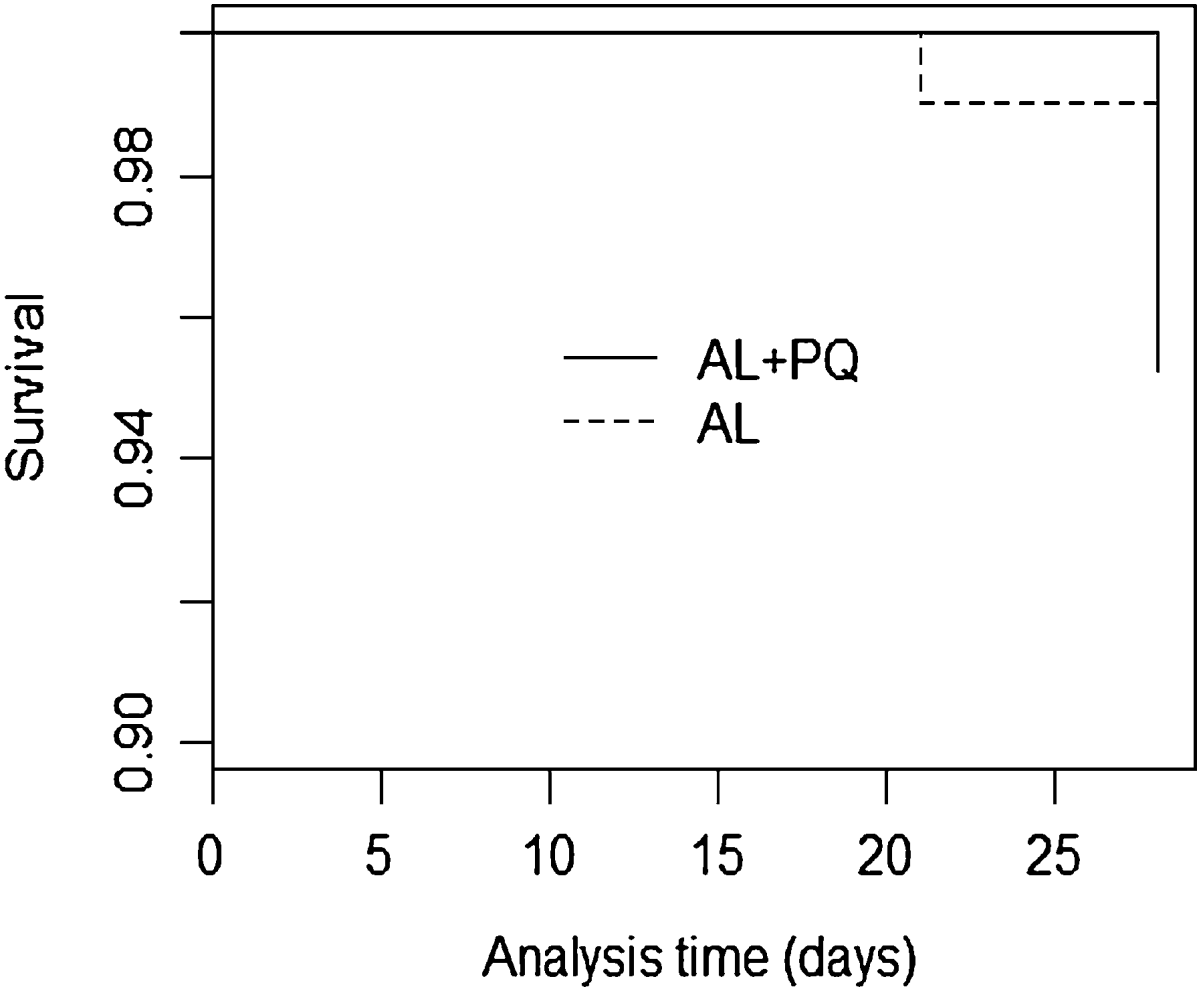
Kaplan-Meier survival curve for cure rate of subjects treated with AL + PQ and AL

Treatment outcomes are presented in Table 2 and Fig. 2. There was no statistically significant difference in the cure (*p* = 0.31) or re-infection rates (*p* = 0.95) by day 28 between the treatment arms. The survival curves for the cure rate were not statistically significant different (*p* = 0.94).

**Table 2:** Treatment outcome

Outcome	Treatment arm		*P*-value
	AL + PQ (n = 107)	AL (n = 104)	
Early treatment failure	0	0	
Late clinical failure	2 (1.8 %)	6 (5.8 %)	0.16
Late parasitological failure	5 (4.7 %)	2 (1.9 %)	0.45
Crude cure rate by day 28	100 (93.5 %)	96 (92.3 %)	0.73
Uncertain PCR outcome by day 28	2 (1.9 %)	2 (1.9 %)	1.0
PCR-determined re-infection rate by day 28	5 (4.7 %)	5 (4.8 %)	0.95
Recrudescence	0	1 (1.0 %)	0.49
PCR-adjusted ACPR by day 28	105 (100.0 %)	101 (99.0 %)	0.31

*ACPR* adequate clinical and parasitological response

### Gametocyte carriage

Microscopic gametocytaemia was only detected in one patient at enrolment and in two patients at 24 h, both treated with AL + PQ. Thereafter, no gametocyte carriage was detected.

### Parasite and fever clearance

**Fig. 3:**
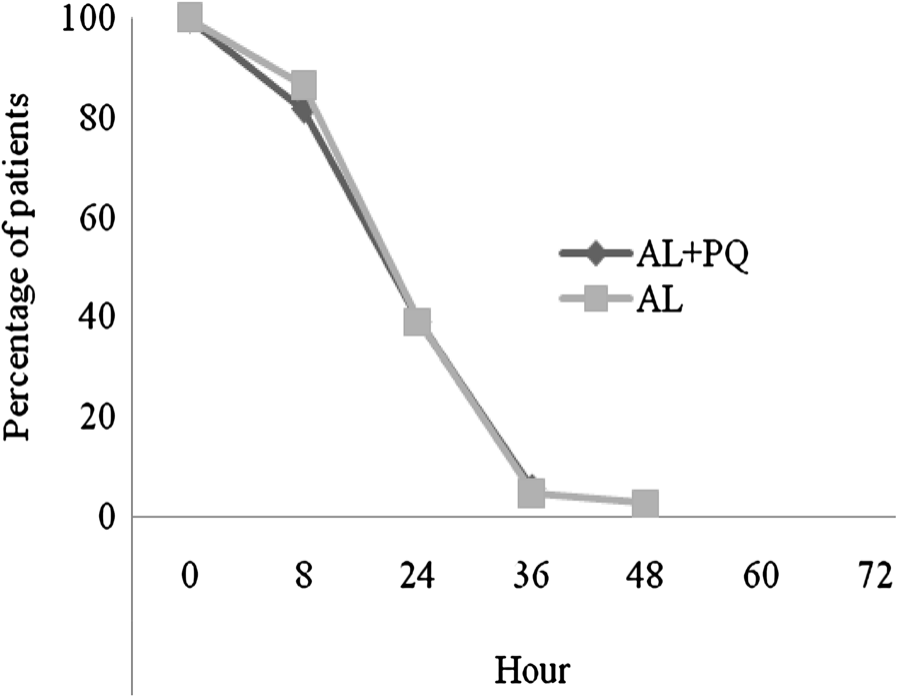
Microscopy-determined parasite clearance following treatment with AL + PQ and AL

**Fig. 4:**
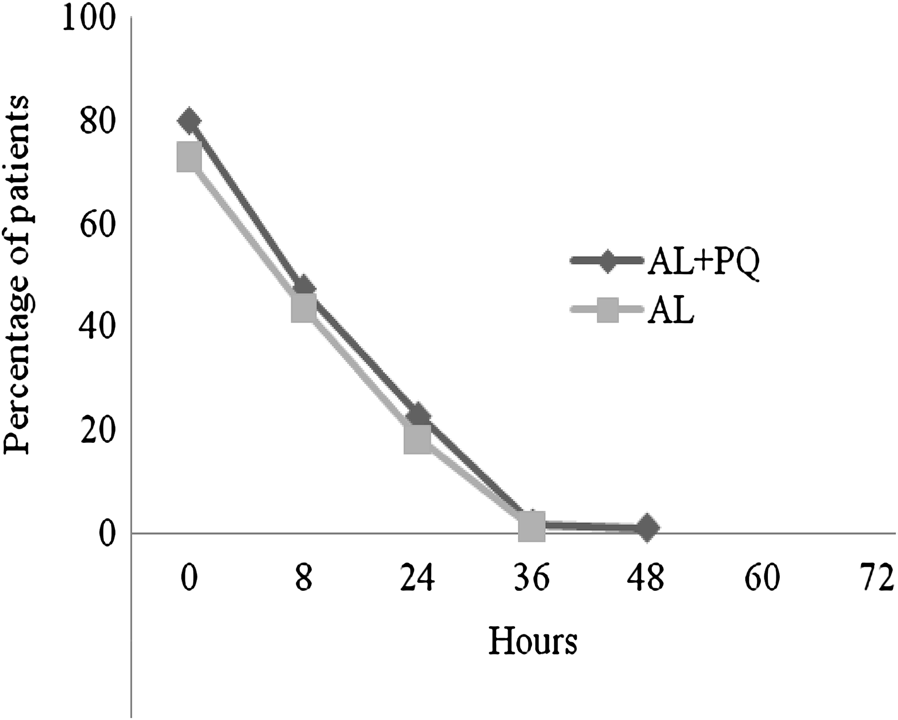
Fever clearance following treatment with AL + PQ and AL

Microscopy-determined asexual parasite and fever clearance was rapid and similar across treatment arms (Figs. 3 and 4). No parasite carriage was observed after 36 and 48 h in AL + PQ and AL arm, respectively.

### PCR detectable parasitaemia on day 3

On day 3, PCR detectable parasitaemia was found in 31/109 (28.4 %) and 29/108 (26.9 %) patients treated with AL + PQ and AL, respectively (*p* = 0.79).

### Prevalence of *Pf*mdr1 N86Y and *Pf*crt K76T

The distribution of *Pf*mdr1 N86Y and *Pf*crt K76T on days 0, 3 and day of recurrent infection across treatments is presented in Table 3. There was neither any statistically significant difference in the proportion of *Pf*mdr1 N86Y or *Pf*crt K76T between treatment arms on days 0, 3 and day of recurrent infection, nor within treatment arms between days 0 and 3 or day 0 and day of recurrent infection. Pooling of all 220 patients did not result in a statistically significant selection in *Pf*mdr1 N86 (*p* = 0.07, McNemar test) or *Pf*crt K76 (*p* = 0.25, McNemar test) between days 0 and 3, or between day 0 and day of recurrent infection [*Pf*mdr1 N86 (*p* = 1.0, McNemar test), *Pf*crt K76 (*p* = 1.0, McNemar test)].

**Table 3:** *Pf*mdr1 N86Y and *Pf*crt K76T distribution on day 0, 3 and day of recurrent infection

		Treatment arm		
		AL+PQ	AL	*P*-value
Day 0				
*Pf*mdr1	N86	91 (85.8 %)	92 (85.9 %)	0.49
	86Y	11 (10.4 %)	8 (7.5 %)	0.45
	N86Y	4 (3.8 %)	7 (6.5 %)	0.54
*Pf*crt	K76	97 (95.1 %)	101 (98.1 %)	0.49
	76T	1 (0.9 %)	0 (0 %)	0.71
	K76T	4 (3.9 %)	2 (1.9 %)	0.44
Day 3				
*Pf*mdr1	N86	15 (65.2 %)	20 (76.9 %)	0.59
	86Y	2 (8.6 %)	2 (7.7 %)	0.54
	N86Y	6 (26.1 %)	4 (15.4 %)	0.48
*Pf*crt	K76	24 (85.7 %)	27 (96.4 %)	0.24
	76T	2 (7.1 %)	0 (0 %)	0.6
	K76T	2 (7.1 %)	1 (3.6 %)	0.6
Day of recurrent parasitaemia				
*Pf*mdr1	N86	2 (66.7 %)	6 (85.7 %)	0.28
	86Y	0 (0 %)	0 (0 %)	–
	N86Y	1 (33.3 %)	1 (14.3 %)	1.0
*Pf*crt	K76	2 (100 %)	4 (80 %)	0.68
	76T	0 (0 %)	0 (0 %)	–
	K76T	0 (0 %)	1 (20 %)	1.0

## Discussion

This study provides much needed data on treatment outcome of the new WHO recommendation of adding a single low-dose (0.25 mg/kg) of PQ to AL compared with standard AL treatment alone. Both regimens provided high cure rates for the treatment of acute uncomplicated *P. falciparum* malaria in Tanzania, with similar PCR-ACPR as previously has been reported from the same study area of AL alone [19–21], and that of other previously reported studies on AL plus 0.75, 0.4 or 0.1 mg/kg single-dose PQ [15, 22]. Similar crude cure rates and PCR-determined re-infection rates were also found between the treatment arms. Moreover, asexual parasite clearance was rapid in both arms. However, a relatively large, but equal, proportion of patients across treatment arms had PCR-detectable parasitaemia on day 3. These findings therefore suggest that the addition of a single low-dose PQ (0.25 mg/kg) does neither compromise the treatment outcome nor interfere with parasite clearance of AL.

Previous studies have reported selection of *Pf*mdr1 N86 and *Pf*crt K76 among recurrent infections during follow-up after AL treatment [23–25], and also between enrolment and day 3 [26]. A similar selection was not observed in the present study. This may be due to that there has been a temporal selection of *Pf*mdr1 N86 and *Pf*crt K76 in the study area ever since AL was introduced as first-line treatment of uncomplicated malaria in 2006 [27], which has resulted in an overall higher baseline prevalence of the two alleles in recent years, which in turn may mask the selection potential of lumefantrine on these drug tolerance/resistance markers. Importantly, adding a single 0.25 mg/kg dose of PQ to AL did not apparently increase the selection potential of AL on *Pf*mdr1 N86 and *Pf*crt K76, neither during the early treatment phase nor among recurrent infections during follow-up after treatment. Thus, the findings suggest that addition of this single low-dose PQ (0.25 mg/kg) does not apparently spur the selection potential of drug resistance markers previously associated with lumefantrine tolerance/resistance.

The study has some potential limitations including that it involved patients of all ages with all levels of parasitaemia despite the WHO recommendation to conduct efficacy studies in children under the age of 5 years with parasitaemia level of 2000–200,000/µl of blood in highly malaria-endemic countries such as Tanzania, to minimize the influence of immunity on treatment outcome in older individuals. However, this study was done to assess whether adding PQ to AL would potentially compromise the efficacy of latter drug probably due to drug–drug interaction, that can be done at all ages and levels of parasitaemia.

## Conclusion

The new WHO recommendation of adding a single low-dose (0.25 mg/kg) of PQ to AL did not compromise treatment outcome of uncomplicated *P. falciparum* malaria in Tanzania. The findings support the use of a single low-dose of PQ together with AL as a potential component of ongoing *P. falciparum* malaria elimination efforts.

## Additional file

10.1186/s12936-016-1430-3 Artemether-lumefantrine + primaquine versus artemether-lumefantrine dataset.

## Abbreviations

ACT: artemisinin-based combination therapy
ACPR: adequate clinical and parasitological response
AL: artemether-lumefantrine
CI: confidence interval
crt: chloroquine resistance
CYP: cytochrome P
DNA: deoxyribonucleic acid
ETF: early treatment failure
GCP: good clinical practices
G6PD: glucose-6-phosphate dehydrogenase
Hb: haemoglobin
IMCH: International maternal and child health
K: lysine
LCF: late clinical failure
LPF: late parasitological failure
mdr: multidrug resistance
N: asparagine
n: sample size
NMCP: National malaria control programme
PCR: polymerase chain reaction
PQ: primaquine
RFLP: restriction fragment length polymorphism
SD: standard deviation
SPSS: statistical package for social sciences
T: threonine
WBC: white blood cell
WHO: World Health Organization
Y: tyrosine
